# Heart Failure: a Major Cardiovascular Complication of Diabetes Mellitus

**DOI:** 10.1007/s11892-016-0809-4

**Published:** 2016-10-12

**Authors:** Gül Bahtiyar, David Gutterman, Harold Lebovitz

**Affiliations:** 1 Division of Endocrinology, State University of New York Health Science Center, Brooklyn, NY USA; 2 Department of Medicine, Woodhull Medical Mental Health Center, Brooklyn, NY USA; 3 Division of Endocrinology, New York University School of Medicine, New York, NY USA; 4 Division of Cardiology, Medical College of Wisconsin, Milwaukee, WI USA

**Keywords:** Diabetes mellitus, Heart failure, Cardiovascular complications

## Abstract

Heart failure (HF) is a major cardiovascular complication of diabetes mellitus (DM). The greatest risk factor for HF is age, and data indicate that 6 to 10 % of individuals over the age of 65 years suffer from HF. Patients with DM have a 2.5-fold increased risk for developing HF than individuals without DM. The 25 to 40 % of patients with HF who have DM have worse outcome (death from cardiovascular disease or hospitalization for worsening HF) than patients without DM. Hyperglycemia is a risk factor for the development of HF with an increase in incidence of HF rising from 10 % at hemoglobin A1c (HbA1c) 8.0 to 9.0 % to 71 % at a HbA1c > 10 %. Patients with DM and HF are equally distributed between those with low ejection fractions and those with normal ejection fractions. The HF treatment regimens for patients with HF and DM (blockade of angiotensin II synthesis or action, cardioselective β-adrenergic blockade, mineralocorticoid receptor blockade, and diuretics) are the same as for HF patients without DM, though the benefit on clinical outcomes is not as great. The new angiotensin-neprilysin inhibitors appear to provide increase outcome benefits in both HF patients with or without DM. Glycemic control impacts the clinical outcomes in patients with HF and DM in a U-shaped relationship with poorer survival at low and high mean HbA1c levels. The optimal chronic glycemic control occurs at an HbA1c of 7.5 to 8.0 % for patients with DM who have symptoms of HF.

## Introduction

Heart failure (HF), a major cardiovascular (CV) complication of diabetes mellitus (DM), has finally emerged as a significant and increasing clinical and public health problem. Several changes in society have coalesced to cause this merger of HF with DM. HF incidence increases with age and is present in 6 to 10 % of individuals 65 years or older [1–3]. This is the most rapidly growing segment of the population in western societies. The lifetime risk of HF at age 55 years is 33 % for men and 28 % for women. The 5-year mortality for persons with HF is approximately 50 %. The prevalence of DM which now is about 415 million persons worldwide is projected to increase by an additional 50 % to 642 million by 2040 [4].

It should not be surprising that the population with both DM and HF is currently between 0.3 and 0.5 % of the total and is growing rapidly. The prevalence of previously diagnosed type 2 diabetes (T2DM) in an HF population is 25 to 35 %, and in more severe hospitalized HF patients, it may be as high as 40 % [5••, 6, 7].

The incidence of HF in patients with clinically diagnosed DM is approximately 2.5 times that in patients without DM [8, 9]. The development of clinical HF in patients with DM is associated with a significantly poorer outcome as measured by CV death or admission to the hospital with worsening HF than comparable non-diabetic subjects [5••, 10–12].

This manuscript focuses on the integration of HF and glycemic management in the increasing population of T2DM and clinical HF to provide preventative and treatment strategies to decrease the prevalence and improve the clinical outcomes for these patients.

### Epidemiology

One of the early reports of the high prevalence of HF in patients with DM (2.5-fold in men and 5-fold in women) compared to non-diabetic individuals came from the Framingham cohort in 1974 [8]. One of the problems in appreciating the importance of HF in patients with DM is the heterogeneity and complexity of developing a mechanistic definition of HF. HF is defined by the American College of Cardiology Foundation (ACCF)/American Heart Association (AHA) guidelines [13] as “a complex clinical syndrome that can result from any structural or functional cardiac disorder that impairs the ability of the ventricle to fill with or eject blood. The cardinal manifestations of HF are dyspnea and fatigue, which may limit exercise tolerance and fluid retention, which may lead to pulmonary congestion and peripheral edema.” HF is classified by the left ventricular ejection fraction (LVEF). The older classification was vague in that HF with preserved LVEF was defined as ≥50 % and with reduced LVEF as ≤40 %, with that between 40 and 50 % arbitrarily assigned one or the other depending on the study design. The more recent classification proposed by the European Heart Association [1] defines LVEF < 40 % as HFrEF (reduced), >40 to 49 % as HFmEF (intermediate), and ≥50 % as HFpEF (preserved). In addition, the diagnosis of HFmEF and HFpEF requires an elevated level of natriuretic peptide and either one or both structural heart disease with left ventricular hypertrophy (LVH) or left atrial enlargement (LAE) or evidence of diastolic dysfunction. The severity of HF is usually defined by the NY Heart Association (NYHA) Classification published in 1964 [14] and/or the ACC/AHA 2009 Guidelines for the Diagnosis and Management of Heart Failure in Adults [13], both of which are provided in Table 1.

**Table 1:** Classification of heart failure

New York Heart Association Classification of Heart Failure [14]	
Class 1	No limitation of physical activity. Ordinary physical activity does not cause undue fatigue, palpitations, dyspnea, or angina pain
Class 2	Slight limitation of physical activity. Patients are comfortable at rest. Ordinary physical activity results in fatigue, palpitation, dyspnea, or angina pain
Class 3	Marked limitation of physical activity. Patients are comfortable at rest. Less than ordinary activity causes fatigue, palpitation, dyspnea, or angina pain
Class 4	Inability to carry on any physical activity without discomfort. Symptoms of heart failure or the angina syndrome may be present even at rest. Any physical activity increases discomfort.
American College of Cardiology/American Heart Association Classification of Heart Failure [13]	
Stage A	High risk for HF but without structural heart disease or symptoms of HF
Stage B	Structural heart disease but without signs of symptoms of HF
Stage C	Structural heart disease with prior or current symptoms of HF
Stage D	Refractory HF requiring specialized interventions

Nichols et al. published a retrospective analysis in 2004 of 8231 patients with T2DM and 8845 non-diabetic patients of similar age and sex who did not have HF at entry in 1997 and were followed for up to 72 months for the development of HF [9]. The patients with T2DM developed 30.9 cases of HF/1000 person-years compared to 12.4 cases/1000 person-years in the non-diabetic individuals (*P* < 0.001). The odds ratio (OR) for HF in all the patients with T2DM as compared to those without DM was 2.5. However, the OR was highly dependent on age: 11.0 at <45 years, 8.6 at 45 to 54 years, 4.4 at 55 to 64 years decreasing to 1.8 at 75 to 84 years. Risk factors for the development of HF in the diabetic population were previous history of ischemic heart disease, poorer glycemic control (hazard ratio (HR) = 1.32 per percentage point of hemoglobin A1c (HbA1c)), and greater body mass index (BMI) (HR = 1.12 per 2.5 units of BMI). Many subsequent studies have confirmed the marked increase in HF in patients with DM compared to non-diabetic individuals [5••, 10, 15, 16]. Data from the Reduction of Atherothrombosis for Continued Health from the (REACH) registry published in 2015 reported results of a 4-year follow-up of 45,227 patients with high-risk atherothrombosis disease of whom 43.6 % had DM at baseline [5••]. In addition to the increase in prevalence of HF observed in the diabetic population, there was also a marked increase in all CV outcomes including death and hospitalization for HF over the 4-year follow-up.

The Organized Program to Initiate Lifesaving Treatment in Hospitalized Patients with Heart Failure (OPTIMIZE-HF) program was a patient registry and performance improvement program for patients hospitalized with HF. The study registered 48,612 patients from 259 hospitals. Forty-two percent of the patients had DM [15].

The incidence of HF in patients with DM increases with poor glycemic control. Data from Kaiser Permanente Medical Care of Northern California reported an 8 % increase in HF risk for every 1 % increase in HbA1c in a population of 48,858 patients with DM followed for a median of 2.2 years [17]. Data from a Swedish National Diabetes Registry of 83,021 patients with T2DM examined the relationship between glycemic control and hospitalization for HF in patients in the registry 1998 to 2003 who were followed up for a mean of 7.2 years [18]. A total of 10,969 (13.2 %) were hospitalized with a primary or secondary diagnosis of HF. Male sex, older age, and longer duration of DM increased the incidence of HF hospitalization (*P* < 0.001). HbA1c was a major risk factor for the development of HF. No increased risk of HF hospitalization was observed with HbA1c < 7 %. A slight increase in risk was observed with HbA1c 7.0 to <8.0 %. The risk for HF hospitalization increased progressively from HbA1c 8.0 to <9.0, 9.0 to <10.0, and >10.0 % (HRs = 1.10, 1.27, and 1.71, respectively, *P* < 0.001).

BMI has been identified as a risk factor for the development of HF in persons with T2DM in several clinical studies [19•, 20]. The Swedish National Diabetes Registry study cited above also determined the effect of BMI on hospitalizations for HF over the same 7.2-year follow-up [19•]. Cox regression analysis of the effect of BMI on HF development adjusting for age, sex, HbA1c, blood pressure, DM duration, smoking, microalbuminuria, cardiac comorbidities, glucose lowering, and anti-hypertensive medications showed the following HR relative to the BMI 20 to 25 group in the 10,969 patients hospitalized for HF: BMI 25 to 27.5 = 1.04, BMI 27.5 to <30 = 1.22, 30 to <35 = 1.54, 35 to <40 = 2.16, ≥40 = 3.22 (*P* < 0.001).

### Pathogenesis of Heart Failure in Patients with DM

HF may occur in the presence of reduced LVEF (HFrEF) or preserved LVEF (HFpEF). The definition of “normal LVEF” has varied over the years. In a 2015 joint guideline from the American Society of Echocardiography and the European Association of Cardiovascular Imaging, “normal LVEF” was defined as being between 53 and 73 % [21]. Popular norms set echocardiographic normative value as LVEF ≥ 55 %; however, in many clinical trials and population-based studies, values as low as ≥40 or 45 % have been considered normal. In addition to LVEF, the patient demography, comorbid conditions, pathogenesis, outcomes, and responses to therapies are somewhat different between the two types of HF [22–25].

#### HFrEF

The primary abnormality in HFrEF is systolic dysfunction in which there is impaired LV contraction and ejection of blood [2, 26–28]. Most common causes are a loss of myocardial mass, impaired myocardial contractility, volume, and/or pressure overload. Hemodynamically, cardiac output is decreased causing hypoperfusion of tissues, end LV systolic and diastolic pressures and volumes are increased, and pulmonary congestion occurs [26–28]. Major factors leading to impaired LV systolic function are coronary ischemic disease and infarction, uncontrolled hypertension, valvular incompetence, and microvascular disease. The decrease in cardiac output and hypoperfusion in HFrEF result in many adaptive responses. Sympathetic nervous system activation causes increases in norepinephrine and epinephrine, activation of the renin-angiotensin-aldosterone system (RAAS), and increases in vasopressin [2, 26–28]. Atrial natriuretic peptide (ANP), ventricular B-type natriuretic peptide (BNP), and C-type natriuretic peptide secretions are increased in response to the elevation in chamber pressure in HF [29]. Cytokines are released from the vasculature and systemic endothelial activation with inflammation results from HF [26, 27]. Chronically, the heart responds to HF with ventricular remodeling resulting in hypertrophy and an increased ventricular volume [2, 26–28]. Many of the adaptive changes (e.g., hypertrophy, heightened sympathetic tone) initially improve ventricular function, but eventually, they become maladaptive and serve to worsen the HF (e.g., chamber enlargement, heightened sympathetic tone). These adaptive changes include molecular remodeling as well. There is reduced expression of SERCA2a, decreased L-type calcium channels on the cardiac myocytes, reduced phosphorylation of phospholamban leading to less SR reuptake of calcium, and altered expression of the sodium-calcium transporter [2, 3, 30, 31]. Treatment of HFrEF is largely focused on blocking or reversing the maladaptive responses and facilitating the adaptive processes [31].

#### HFpEF

HF with preserved EF has been defined by the European Society of Cardiology using the following criteria: (1) signs or symptoms of HF, (2) normal or mildly abnormal systolic LV function, (3) elevated levels of natriuretic peptides and at least one of the two following criteria: relevant structural heart disease (LVH and/or LAE) or diastolic dysfunction [1]. Normal or mildly abnormal systolic LV function was defined as an LVEF ≥ 50 % and an LV end diastolic volume index (LVEDVI) < 97 ml/m^2^. Diastolic LV dysfunction is considered as LV end diastolic pressure >16 mm Hg or mean pulmonary capillary wedge pressure > 12 mm Hg [23]. The AHA and ACC define HFpEF simply as HF in which the EF > 50 % [32]. Many observers continue to equate HFpEF with diastolic dysfunction. The underlying abnormalities in most cases of HFpEF are increased LV stiffness, impaired LV relaxation, impaired contractile reserve, increased vascular stiffness and inefficient ventriculo-arterial coupling, pulmonary hypertension, and abnormal cardiovascular response to exercise. Emerging evidence points to microvascular dilatory dysfunction or rarefaction as a significant etiologic factor in the development of diastolic dysfunction [22, 33–35]. The prevalence of HFpEF is greater in women, older age populations, and in those with hypertension.

Patients with DM are at high risk for both HFrEF and HFpEF [11, 36, 37]. Approximately 40 % of diabetic subjects with HF have HFpEF which is similar to that in non-diabetic subjects with HF. However, the overall incidence of HF in diabetic subjects is 2.5-fold greater, and clinical outcomes are significantly worse than in the non-diabetic population [11, 38]. What factors can explain these disparities? Ischemic heart disease and hypertension are comorbidities with DM and are major risk factors for HF. However, numerous studies have shown that DM itself is an independent risk factor for HF [17, 39••, 40, 41]. Diabetic cardiomyopathy is a clinical condition in which ventricular dysfunction occurs in DM patients in the absence of coronary artery disease (CAD) or hypertension [42–44]. It is often difficult to isolate the contribution of cardiomyopathy from the other risk factors such as CAD and hypertension because of their frequent co-existence. In a nationwide study of hospital discharges of 44,837 patients with unexplained dilated cardiomyopathy and 450,254 matched controls, DM was significantly more prevalent in the HF group (adjusted OR = 1.58, 95 % CI = 1.55–1.62) [45]. One potential reason for the occurrence of HF in patients with DM independent of CAD or hypertension is the associated microvascular dysfunction characteristic of DM. Impaired reactive hyperemia and other measures of microvascular dysfunction are observed in DM and could explain the rate of HFpEF [34, 35]. An early feature of diabetic cardiomyopathy is diastolic dysfunction.

#### Diabetes

DM leads to an increase in myocardial free fatty acid (FFA) uptake and metabolism and a reduction in glucose metabolism. The excessive myocardial FFA uptake and metabolism are thought to lower LV diastolic and systolic function, increase mitochondrial reactive oxygen stress (ROS), and cause additional lipotoxic effects such as inflammation in myocardial cells [39••, 40, 44]. Hyperglycemia elevates cardiomyocyte glucose levels which generate advanced glycosylation end-products (AGEs) and glucose toxicity. AGEs increase ROS, can crosslink and damage collagen, and activate the inflammatory cascade among its many effects. The net effect of the multiple metabolic effects of DM on the myocardium is to cause myocardial hypertrophy, myocardial fibrosis and stiffness, myocardial microangiopathy, and impaired myocardial contractility [25, 42].

If diabetic cardiomyopathy is due to microangiopathy and the metabolic consequences of poorly regulated hyperglycemia, it should be possible to prevent it by early and aggressive management of the lipid and glucose abnormalities of DM [46].

### Clinical Course of Heart Failure in Patients with Diabetes

The prognosis of patients with HF is poor. Community-based studies indicate that 1-year mortality following diagnosis is 30 to 40 % and 5-year mortality is 40–60 %. Refractory HF or sudden ventricular arrhythmia are the cause of death [47]. The NYHA classification gives useful prognostic information for an individual patient. Patients with NYHA class IV have a 30 to 70 % annual mortality while those with class II have an annual mortality of 5 to 10 % [48].

Patients with DM and HF have a poorer outcome than non-diabetic patients. In the REACH Registry of 45,227 HF patients, 43.6 % of whom had DM, the 4-year HR of cardiovascular death, non-fatal MI or non-fatal stroke in the diabetic population (16.5 %) compared to the nondiabetic (13.1 %) was 1.27, *P* < 0.001. CV death occurred in 8.9 versus 6.0 % (*P* < 0.001) and overall death 14.3 versus 9.9 % (*P* < 0.001) (DM versus non-diabetic individuals). DM patients had a 33 % greater rate of hospitalizations for HF, 9.4 vs 5.9 % (*P* < 0.001). In the patients with HF at baseline, the HR for cardiovascular death in the diabetic population was 2.45, and for hospitalization for HF, it was 4.72 (*P* < 0.001) [5••].

An analysis of the impact of DM on outcomes in patients with HFrEF and HFpEF performed in 7599 patients with symptomatic HF followed for a median of 37.7 months in the Candesartan in Heart failure-Assessment of Reduction in Mortality and morbidity (CHARM) program showed that DM was an independent predictor of CV mortality and morbidity regardless of EF. The prevalence of DM was 28.3 % in those with preserved EF and 28.5 % in those with reduced EF. For the entire population of 2163 participants with DM, the rates of death and hospitalizations from CV disease (94.4 and 306.5 per 1000 patient years of follow-up, respectively) were greater than those for the 5436 non-diabetic population (57.5 and 207.4 per 1000 patient years of follow-up, respectively, *P* < 0.001). The patients with DM and preserved EF had worse outcomes (CV death and HF hospitalization) relative to those without DM who had HFpEF (HR = 2.0, *P* < 0.0001). In participants with DM and low EF, DM was an independent predictor of CV death or HF hospitalization with an HR of 1.60 (*P* < 0.0001) compared to non-diabetic individuals [11]. In the Digitalis Investigation Group (DIG) ancillary study which compared participants with DM and HFpEF to participants without DM but with HFpEF, DM was associated with a 68 % increased risk of HF hospitalization or HF death during a mean follow-up of 37 months [37].

Several studies have assessed the impact of fasting plasma glucose on outcomes of patients with HF. A retrospective analysis of the effect of fasting plasma glucose on outcomes in 6607 patients with HF followed in a Jerusalem Health Maintenance Organization indicated that impaired fasting plasma glucose (IFG) and elevated plasma glucose (DM) predict reduced survival (HR = 1.42 for DM; 1.55 for IFG, *P* < 0.01 for both) and increased cardiac-related hospitalizations (HR = 1.31, *P* < 0.001 for DM; 1.17, *P* < 0.05 for IFG) when compared to HF patients with fasting glucoses 92 to 99 mg/dl. Forty-eight percent of the patients had DM and 11 % had IFG. The mean follow-up was 487 days, and the mean age at inclusion was 75 ± 13 years [49].

Undiagnosed DM is a frequent occurrence in patients hospitalized for HF. In a series of 400 patients admitted consecutively for acute HF, 47 % did not have DM, 37 % had known clinical DM, and 16 % were previously undiagnosed with DM but had two fasting plasma glucoses ≥7.0 mmol/l before or after the acute episode. Despite having comparable CV risk factors, the patients with undiagnosed DM had a 7-year total and CV mortality that was significantly higher than those without DM (HR = 1.69, 95 % CI = 1.17–2.46 and 2.45, 95 % CI = 1.58–3.81, respectively) and similar to those with known DM who had a much higher CV risk profile [50]. The Worcester Heart Failure Study (WHES) was a population-based surveillance study of adult residents of the Worcester, MA metropolitan area admitted to all 11 central Massachusetts medical centers for acute decompensated HF during the years 1995, 2000, 2002, and 2004. The total population surveyed consisted of 5428 individuals without DM (glucose 124.8 ± 28 mg/dl), 3807 with diagnosed DM (glucose 191 ± 96 mg/dl), and 513 with admission hyperglycemia (glucose 260.5 ± 65 mg/dl). The highest in-hospital death rate occurred in those with admission hyperglycemia (9.9 %) compared to 6.5 % in patients with known DM and 7.5 % in individuals without DM [51•]. This is consistent with the observations that patients admitted with an acute illness who have severe hyperglycemia have previously unrecognized and uncontrolled DM or have a severe illness that causes markedly excessive neuroregulatory and cytokine responses [52].

The Meta-Analysis Global Group in Chronic Heart Failure (MAGGIC) reported an analysis of individual data on 39,372 patients with both HFrEF and HFpEF from 30 cohort studies. A total of 40.2 % of the patients during a median follow-up of 2.5 years died. They constructed a model from the data to predict mortality from HF. The model includes 13 independent predictors of mortality. The order of predictive strength is age, lower EF, NHYA class, serum creatinine, DM, absence of prescribed beta-blocker, lower systolic blood pressure, lower body mass, time since diagnosis, current smoker, chronic obstructive pulmonary disease, male gender, and absence of a prescribed RAAS inhibitor [53]. In HFpEF, age was more predictive, and systolic blood pressure was less predictive of mortality than in HFrEF. The Web site address for the MAGGIC calculator for all-cause mortality for 1 and 3 years is http://www.heartfailurerisk.org. An alternative predictive model for survival in HF is the Seattle Heart Failure Model [54].

## Treatment of Heart Failure in Patients with Diabetes

The treatment of HF in the patient with DM is similar to the individual without DM with the added complication of the impact that glycemic control and the anti-hyperglycemic agents used have on clinical HF outcomes. In both the diabetic and non-diabetic populations, treatment for HF with reduced LVEF (<40 %) provides a significant benefit in reducing total mortality, CV mortality, and hospitalization for progressive HF. In contrast, these same therapies do not result in improved survival in patients with HF with preserved LVEF (>45 or 50 %).

### Patients with HFrEF–LVEF ≤ 40 %

#### Blockade of the RAAS

In patients with HFrEF, therapy is directed toward reversing the maladaptive effects that occur because of the activation of the sympathetic nervous system and the RAAS. Angiotensin converting enzyme inhibitors (ACEI), which block the conversion of angiotensin I to angiotensin II, reduce total mortality 23 % and the combined end point of mortality or hospitalization for HF 35 % [55]. The effects are similar among the different ACEI [56–59]. Patients with the lowest EF appear to have the greatest benefit with the greatest effects occurring in the first few months of treatment. The reduction in mortality is due to fewer deaths from progressive HF. ACEI have little if any effect on sudden, arrhythmic death. ACEI block the production or metabolism of many peptides other than angiotensin II. Their use is associated with significant side effects such as cough, angioedema, symptomatic hypotension, and renal dysfunction.

Because angiotensin II receptor blockers (ARBs) effectively reduce angiotensin II effects without the side effects of ACEIs, their value in treating HF has been evaluated in many clinical trials. The initial trials examined the effects of adding an ARB to patients with HFrEF on standard care including ACEIs and to HFrEF patients unable to take ACEIs because of intolerance to their side effects. Valsartan added to HFrEF patients NYHA class II–IV receiving standard care (93.5 % on ACEIs, 35 % on β-adrenergic blockers, 5 % on spironolactone) did not reduce overall mortality but did decrease the combined endpoint of mortality and hospitalization for progressive HR by 13.2 % [60]. This was primarily due to the lower number of patients hospitalized for HF (18.2 % in the placebo-treated group versus 13.8 % in the valsartan-treated group, *P* < 0.001). A post hoc analysis of the data suggested that adding valsartan to patients taking both an ACEI and a β-adrenergic blocker increased mortality (*P* = 0.009) and showed a tendency toward a poorer combined outcome as compared to placebo treatment. The post hoc analysis also suggested that the effect of valsartan on the combined endpoint was not significant in the 25 % of the patients with HF and DM. The response to valsartan addition appeared to be somewhat better in the 366 patients not taking an ACEI [61]. Compared to the placebo, both all-cause mortality and combined all-cause mortality and CV morbidity in the valsartan group were significantly less (17.3 versus 27.1 % and 24.9 versus 42.5 %, respectively). It appeared that an ARB could replace an ACEI with at least equal benefits.

A comparable series of studies with candesartan in patients with HFrEF and NYHA class II–IV yielded slightly different results. Candesartan added to patients taking ACEIs 100 %, β-adrenergic blockers 55 %, and spironolactone 17 %, decreased CV death or hospital admission for HF over a median duration of treatment of 41 months by 15 % (38 % events in candesartan group versus 42 % in placebo group, *P* = 0.01) [62]. CV deaths were reduced by 16 % and hospitalization for HF by 17 %. In this study, patients on ACEIs and β-adrenergic blockers had the same benefits from candesartan as all other patients. Adding candesartan to patients with HFrEF not taking ACEIs because of intolerance resulted in a 23 % reduction on CV death and hospitalization for HF, a 15 % reduction in CV death, and a 32 % reduction in hospitalization for HF [6]. Adding an ARB to an ACEI increases the incidence of increasing renal dysfunction and hyperkalemia [6, 61]. The combination ACEI and ARB blocker does not appear to provide significant clinical benefit but is associated with more significant side effects.

Blocking the RAAS improves clinical outcomes in patients with HFrEF. The magnitude of the benefit depends on sufficiently blocking the RAAS and is therefore dependent on the dose of the inhibitor or blocker used. There is no clear data as to the effectiveness of RAAS inhibition on HF outcomes in patients with DM as compared to those without DM.

#### Renin Inhibition

A large clinical trial examined the effect of a renin inhibitor (aliskiren) compared to enalapril or combined with enalapril on the primary outcome of death from CV causes or hospitalization for HF. Participants were randomized to enalapril 5 or 10 mg twice a day (*n* = 2336), aliskiren 300 mg once daily (*n* = 2340), or enalapril + aliskiren (*n* = 2340). After a median follow-up of 36.6 months, the primary outcome occurred in 32.0 % of the combined therapy group, 34.6 % in the enalapril group, and 33.8 % in the aliskiren group. Aliskiren alone or combined with enalapril provided no addition benefit on the clinical outcomes. The combination of aliskiren and enalapril increased the risk of hypotensive symptoms, an elevated serum creatinine, and hyperkalemia [63].

#### B-adrenergic Blockade

B-adrenergic blocking agents improve functional status and reduce morbidity and mortality in patients with HFrEF. In order to minimize adverse effects such as impaired recognition of clinical hypoglycemia, deterioration of glycemic control, elevation of lipids, bronchospasm, and inhibition of vasodilator effects of β2-adrenergic stimulation, cardio-specific β1-receptor antagonist are recommended. Bisoprolol administered for a mean of 1.3 years to patients with NYHA class III and IV HF with an EF ≤ 35 % receiving standard therapy with diuretics and ACEIs reduced all-cause mortality by 34 % and sudden deaths by 44 % compared to placebo treatment (*P* < 0.0001) [64]. Metoprolol CR/XL in the Metoprolol CR/XL Randomized Intervention Trial in-Congestive Heart Failure (MERIT-HF) trial involving 3991 participants with NYHA class II-IV and EF ≤ 40 % on standard therapy reduced all-cause mortality at 1 year of follow-up by 34.5 % compared to placebo (*P* < 0.001). Sudden death was reduced by 41 % and death from worsening HF by 49 %. The metoprolol dose was started at 12.5 or 25 mg daily and up-titrated over 8 weeks to 200 mg daily [65]. Similar results were obtained by carvedilol treatment of patients with severe chronic HF (symptoms at rest or with minimal exertion and an EF < 25 %). In the carvedilol prospective randomized cumulative survival (COPERNICUS) study, after an average treatment of 10.8 months, there were 190 deaths in the placebo-treated group and 130 in the carvedilol group for a risk reduction of 35 %, *P* < 0.001. Carvedilol reduced the combination of CV death or hospitalization for HF by 31 %, *P* < 0.001 [66, 67]. The carvedilol-treated participants spent 27 % fewer days in the hospital and experienced a serious adverse event like sudden death, cardiogenic shock, or ventricular tachycardia less than placebo-treated participants (*P* < 0.002). β-adrenergic blockade has dramatic effects in reducing mortality and sudden death in particular. Metoprolol succinate, carvedilol, and bisoprolol are the currently marketed β-adrenergic blockers in the USA shown to exert a mortality benefit in HFrEF.

#### Mineralocorticoid Receptor Antagonists

In clinical trials, the mineralocorticoid receptor antagonists, spironolactone and eplerenone, have been shown to increase survival among patients with severe systolic HF. In the Randomized Aldactone Evaluation Study, 25 mg of spironolactone and placebo were administered to 1663 participants who had severe HF and a LVEF ≤ 35 % and were being treated with an ACEI, a loop diuretic, and in most cases digoxin. The primary endpoint was death from all causes. The study was terminated early after a mean follow-up of 24 months. Forty-six percent of participants died in the placebo arm and 35 % in the spironolactone arm, for a risk reduction (RR) of 30 %, *P* < 0.001. The risk reduction in the spironolactone-treated group was attributed to both a reduction in sudden death from cardiac disease and a decrease in death due to progressive HF [68]. Participants who received spironolactone had a 35 % lower frequency of hospital admission for worsening HF (*P* < 0.001) and an improvement in NYHA functional class (*P* < 0.001). Similar results were observed in a NYHA class II population treated with eplerenone (up to 50 mg/day) for a median duration of 21 months. A composite end point of death from a CV cause or hospitalization for HF occurred in 25.9 % of the placebo group and 18.3 % in the eplerenone group for an RR of 37 % (*P* < 0.001) [69]. All-cause mortality and CV mortality were decreased 34 % (*P* < 0.01). A serum potassium level exceeding 5.5 mmol/l occurred in 11.8 % of the eplerenone-treated participants and 7.2 % in the placebo-treated participants. Use of a mineralocorticoid receptor antagonist is accepted as standard of care for symptomatic patients with HFrEF (EF < 35 %) who are also taking beta-adrenergic blockers and either ACEI or ARB. Monitoring serum potassium levels should be done routinely.

#### Angiotensin-Neprilysin Inhibition

One of the physiologic adjustments to HF is an increase in the secretion of atrial natriuretic hormone and BNP. These hormones are released in response to myocardial stretching in the atria and the ventricles and induce diuresis, natriuresis, and vasodilatation as well as inhibiting the sympathetic nervous system [29, 70]. Attempts to treat HF with recombinant human BNP were unsuccessful with several clinical trials showing no significant beneficial effects and some possible toxic effects [29, 70]. An approach which has been successful is the development of agents to block the enzyme neprilysin that metabolize NPs and increase endogenous NP levels [71]. One such agent is AH 377 (sacubitril), a prodrug that is rapidly metabolized into a biologically active neprilysin inhibitor. This was coupled to valsartan, and the angiotensin-neprilysin (sacubitril/valsartan) inhibitor has been shown in the PARADIGM-HF trial to be more effective in treating HF than enalapril [7]. The combined inhibitor (LCZ696) at 200 mg twice a day was compared to 10 mg enalapril twice a day in a double-blind, randomized trial involving 8442 participants with NYHA HF class II, III, or IV and a LVEF ≤ 40 %. The trial was stopped early after a median follow-up on 27 months. The primary outcome, a composite of death from CV causes or hospitalization for HF, occurred in 21.8 % of the LCZ696-treated participants and 26.5 % of the enalapril-treated participants (RR = 20 %, *P* < 0.001). The RR for all-cause mortality was 16 %, for CV death 20 %, and for hospitalizations for HF was 21 %.

A recent analysis of the PARADIGM-HF data examined the risk related to pre-diabetes and DM [12]. Patients with a baseline history of DM (*n* = 2907, 35 %) had a higher risk of the primary composite outcome of HF hospitalization or CV mortality compared to those without a history of DM (*n* = 5492), (adjusted HR = 1.38, *P* < 0.001). Baseline HbA1c measurements showed that an additional 1106 (13 %) participants had undiagnosed DM (HbA1c ≥ 6.5 %) and 2103 (25 %) had pre-diabetes (HbA1c 6.0–6.4 %). The HRs for the composite primary outcome in participants with previously known DM, those with previously unknown DM, and those with pre-diabetes compared to participants without DM (HbA1c < 6.0 %) were 1.64, 1.39, and 1.27, respectively, *P* < 0.001 for all three groups. In this large clinical trial which randomly recruited participants with reduced LVEF, 49 % of the participants had DM and 25 % had pre-diabetes. The primary clinical outcomes over 27 months were increasingly worse going from normoglycemia to pre-diabetes to newly diagnosed DM to known DM. Some benefit of LCZ696 (sacubitril/valsartan) treatment compared to enalapril treatment on clinical outcomes was observed irrespective of glycemic status. The HRs for the primary composite outcome comparing sacubitril/valsartan to enalapril treatment going from normoglycemic to pre-diabetes to newly diagnosed DM to known DM were 0.68 to 0.76 to 0.97 to 0.87. The occurrence of DM in the patient with HF results in worse clinical outcomes and poorer responses to current medical therapy.

#### I_f_ Current Blockade

Ivabradine blocks the I_f_ current responsible for automaticity in the sinoatrial node of the heart. Its primary action is to slow heart rate without affecting ventricular myocardium. In patients with persistently elevated heart rates despite the use of β-adrenergic blockers, Ivabradine can slow the heart rate, improve left ventricular filling, and reduce mortality. The SHIFT trial (Ivabradine in addition to beta blockade therapy for participants with mild to moderate HF and heart rates above 70 bpm) showed that the 2-year composite outcome of cardiovascular death or hospital admission was less in the group treated with Ivabradine than in those randomized to placebo (HR = 0.74, *P* < 0.001) [72]. In a post hoc analysis of the participants in the SHIFT trial, the prevalence of DM in the population was 30 % of whom 32 % were being treated with insulin. The participants with DM had an increase in the primary outcome of CV death or hospitalization for worsening HF compared to the non-diabetic population (HR = 1.18, *P* = 0.001) [73]. The beneficial effects of Ivabradine occurred in the diabetic population (HR = 0.80) as well as the non-diabetic population (HR = 0.84).

#### Other Medical Therapies Not Shown to Have a Mortality Benefit

In patients with HFrEF NYHA classes II–IV, about 85 % of patients ordinarily require treatment with diuretics, and loop diuretics are preferred. Approximately 50 % of patients with HFrEF are administered a digitalis preparation. Clinical trials have shown that digoxin has no effect on overall mortality but does cause a reduction in the rate of hospitalization for overall and worsening HF in patients with HFrEF [74]. In an ancillary study, digoxin had no effect on either mortality or morbidity in patients with HFpEF [75]. Both diuretics and digitalis are used primarily for symptomatic improvement; however, the therapeutic window for digitalis is rather narrow, limiting its clinical utility.

#### Non-surgical Device Treatments

When medical therapy fails after an appropriate trial, medical device therapy can be considered. Implantable cardioverter-defibrillator (ICD) is useful for both primary and secondary prevention of sudden death from ventricular arrhythmia. Cardiac resynchronization therapy (CRT) can be considered in symptomatic patients with HF in sinus rhythm with a QRS ≥ 150 msec with or without left bundle branch block morphology and an EF ≤ 35 % to improve symptoms and reduce morbidity and mortality. Device therapy requires evaluation and chronic care by cardiologists with specific expertise in the use of these devices.

A device approved in European countries and still in clinical trials in the USA is cardiac contractility modulation (CCM) which involves non-excitable electrical stimulation of the ventricles during the absolute refractory period to enhance contractile performance.

There is no information as to the benefit of these devices in the patient with HF with DM as contrasted to subjects without DM. A detailed discussion of device therapy for HF can be obtained in the 2016 ESC Guidelines for the diagnosis and treatment of acute and chronic HF [1].

### Patients with HFpEF–LVEF > 40 %

Though much knowledge and understanding about the pathophysiology of HFpEF has been obtained in the last decade, there is, as yet, no treatment identified that provides a proven mortality benefit. Several clinical trials suggest a possible benefit in morbidity and a large trial with ARB/neprilysin inhibition is ongoing.

#### Angiotensin Receptor Blockade

The CHARM Preserved Trial recruited 3023 participants with NYHA classes II–IV and LVEF > 40 %. Median follow-up was 36.6 months, and the primary outcome was CV death or hospital admission for HF. Patients were randomized to candesartan 32 mg once daily or placebo. Of the cohort, 22 % of the candesartan and 24 % on the placebo group experienced the primary outcome (HR 0.89, *P* = 0.118). There was no difference in CV death (170 participants in each group), but fewer participants on candesartan had a hospital admission for HF (230 versus 279, *P* = 0.017) [76]. A trial involving 4128 participants (≥60 years) with NYHA class II–IV, LVEF ≥ 45 % randomized subjects to irbesartan versus placebo. After a mean follow-up of 49 months, there was no significant difference between the two groups in primary outcome of death from any cause or hospital admission for a CV cause [77].

The paramount trial was a phase II trial in participants with NYHA class II–IV, LVEF ≥45 %, and NT-proBNP > 400 pg/ml to evaluate the effect of LCZ696 200 mg twice a day versus valsartan 160 mg twice daily on NT-proBNP levels at 12 weeks. LCZ696 lowered the NT-pro BNP levels by 4 weeks and maintained those levels through 36 weeks [78]. Valsartan caused a slow progressive decrease in NT-proBNP during the entire 36 weeks. The improvement by LCZ696 treatment over valsartan was significantly different at 4 and 12 weeks, but not at 36 weeks. LCZ696 caused a greater BP reduction at 12 and 36 weeks. The preliminary data from this pilot study was used to help design the large ongoing PARAGON-HF in HFpEF study which will study the effect of LCZ676 on clinical outcomes in participants with HFpEF.

## Treatment of Hyperglycemia in Patients with Diabetes and Heart Failure

Patients with T2DM have a marked increase in the prevalence of HF and have a poorer prognosis when they develop HF than non-diabetic individuals. A major consideration is the extent to which hyperglycemia itself contributes to these disparities. Hyperglycemia may impact HF in several ways: (1) chronic hyperglycemia may contribute to the development of HF, (2) hyperglycemia may worsen the outcome of acute HF, (3) chronic hyperglycemic management in the patient with chronic HF may influence clinical outcomes, (4) specific anti-hyperglycemic agents may have direct effects on the development and/or clinical outcomes of HF.

As noted in the section on epidemiology, observational studies have shown a striking relationship between HbA1c and the development of HF in patients with T2DM and no HF at baseline. During a mean follow-up of 2.2 years, each 1 % increase in HbA1c was associated with an 8 % increase in HF and for a mean follow-up of 7 years, HF development increased progressively from <5 % with an HbA1c of 7.0 to 72 % with an HbA1c greater than 10 % [18].

The level of chronic glycemic control and the stage of HF determine the clinical outcomes of CV death and hospitalization for HF in patients with DM. The large clinical intervention trials in participants with T2DM to determine the effect of intensive (HbA1c 6.5 to 6.8 %) versus ordinary glycemic control (HbA1c 7.5 to 8.0 %) on CV events failed to show any effect on HF outcomes (HR = 1.00, 95 % CI = 0.86–1.16) [79]. While these trials included 27,049 participants, the baseline incidence of HF was very low (approximately 5 %), and only 905 participants were hospitalized for HF annually. The overall difference in mean HbA1c between the groups in all of those intervention trials was 0.9 %.

In contrast, observational studies in patients with HF and DM show a strong relationship between glycemic control and clinical outcomes. In a population-based study of 16,524 patients seen in emergency departments in Ontario, Canada with acute HF syndromes, the presenting blood glucose level predicted clinical outcomes [80]. A presenting blood glucose >11.1 mmol/l in patients with DM (*n* = 7249) was associated with an increased risk of all-cause death (HR = 1.48, *P* = 0.01) or DM-related hospitalizations (HR = 1.39, *P* < 0.001). A presenting blood glucose >9.4 mmol/L was associated with an increase in risk of hospitalization for HF (HR = 1.15, *P* = 0.002) or any CV cause (HR = 1.09, *P* = 0.009) in the entire patient population [80]. The Worcester WHES study observed the effects of elevated serum glucose levels on survival after acute HF in 5428 non-diabetic patients, 3807 patients with diagnosed DM, and 513 patients with admission hyperglycemia. In-hospital death rate was highest in those with admission hyperglycemia (9.9 %) as compared to individuals without DM (7.5 %) and those with known DM (6.5 %). In contrast, the patients with known DM had the highest death rates 3 months (HR = 1.14 compared to those without DM, 95 % CI = 1.09–1.20) and 1 and 2 years (HR = 1.18, 95 % CI = 1.11–1.24) after discharge [51•].

The ASTRONAUT trial (aliskren) [81] and the EVEREST trial (tolvaptan) [82] were clinical trials in participants hospitalized for symptomatic HF. In both studies, the participants were assessed for post-discharge outcomes, and differences were determined in participants with known DM as compared to participants without DM. The extent of glycemic control was not monitored by glucose levels or HbA1c in either study. ASTRONAUT trial showed no difference in inpatient outcomes between participants with and without DM. However, 12 months after discharge, participants with DM had higher CV mortality or hospitalization for HF (non-diabetic HR = 0.80; diabetic HR = 1.15, *P* < 0.03). A mean of 9.9 months after discharge, participants with DM in the EVEREST trial had a 17 % increase in CV mortality or hospital admission for HF. Patients with DM in the EVEREST trial were treated by diet (20 %), oral agents (36 %), or insulin (48 %). The insulin-treated group had an increase in the outcome of CV mortality or HF hospitalization (HR = 1.25, 95 % CI = 1.00–1.57) compared to the other treatments. The increase in outcome events in the short-term post-discharge period of these two studies suggests an influence of glycemic control on outcomes in participants with DM and HFrEF.

Several retrospective studies have examined the effect of the intensity of glycemic control on mortality in patients with DM and chronic HF. Table 2 summarizes the results from three such studies, and all indicate that there is a U-shaped relationship between mortality and HbA1c levels [83–85]. The most comprehensive study examined the mortality during a 2-year follow-up period from the database records of 5815 patients with DM and HF managed in ambulatory clinics in VA Medical Centers [83]. Ninety-four percent of patients were male with a mean age of 69.2 years; 45 % had reduced LVEF and 55 % had preserved LVEF. The other two studies were each from a single center, included only patients with reduced LVEF, and reported data from a 2-year follow-up [84, 85]. The patients in the Tomova et al. study had more severe HF with 75 % NYHA class III and IV [84]. The data from the three studies suggest that the lowest mortality occurs when glycemic control maintains the HbA1c between 7.5 and 8.0 % (Table 2).

**Table 2:** Glycemic control and mortality in patients with heart failure and diabetes

Aguilar et al. [83]		Tomova GS et al. [84]		Eshaghian S et al. [85]	
*N* = 5815	Mortality at 2 years	*N* = 358	Mortality or urgent heart transplantation at 2 years	*N* = 123	Mortality at 2 years
Quintile 1 HbA1c ≤ 6.4 %	25 %	Quartile 1 HbA1c ≤6.4 %	52 %	Quartile 1 HbA1c <6.6 %	9/31 29 %
Quintile 2 HbA1c 6.4 to ≤7.1 %	23 %	Quartile 2 HbA1c 6.5 to 7.2 %	58 %	Quartile 2 HbA1c 6.6 to 7.7 %	13/33 39 %
Quintile 3 HbA1c 7.1 to ≤7.8 %	17.7 %	Quartile 3 HbA1c 7.3 to 8.5 %	39 %	Quartile 3 HbA1c 7.8 to 8.9 %	3/29 10 %
Quintile 4 HbA1c 7.8 to ≤9.0 %	22.5 %	Quartile 4 HbA1c ≥8.6 %	35 %	Quartile 4 HbA1c >8.9 %	7/30 23 %
Quintile 5 HbA1c > 9.0 %	23.2 %				

An additional issue in treating the patient with DM and HF is the choice of anti-hyperglycemic agents used. There are no adequate clinical trials of metformin, sulfonylurea, or ordinary insulin dose on HF pathogenesis or treatment. The only data available are from observational studies. A retrospective analysis of 6185 patients with HF and DM treated in ambulatory clinics in Veterans Affairs medical centers and followed for 2 years showed a propensity score adjusted mortality in metformin-treated patients of 16.1 versus 19.8 % in patients not treated with metformin (HR = 0.76, *P* < 0.01). HF hospitalization was no different between metformin treatment and no metformin treatment [86]. Based on the US and Canadian cardiac failure data, treatment with metformin is not absolutely contraindicated in patients who have isolated HF. The risk of lactic acidosis due to metformin is negligible in these patients and is unrelated to the plasma concentration of metformin. Metformin-associated lactic acidosis may occur when kidney function is decreased in patients with decompensated HF. Metformin provides a greater degree of CV protection and should not be withheld in patients with DM with stable HF who do not have other risk factors for acute decompensated HF or lactic acidosis [87].

In a single center observational study of 554 consecutive patients with advanced systolic HF, patients were stratified into 3 groups: 43 patients with DM were treated with insulin and 89 were non-insulin-treated. The 1-year survival was 89.7 % in the 422 patients without DM, 85.8 % in the non-insulin-treated patients with DM, and 62.1 % for the insulin-treated patients with DM, *P* < 0.00001. After Cox multivariate analysis, insulin-treated DM was found to be an independent predictor of mortality (HR = 4.30, 95 % CI = 1.69–10.94) whereas non-insulin-treated DM was not (HR = 0.95, 95 % CI = 0.31–2.93) [88]. A large CV outcome trial (ORIGIN) comparing low-dose basal insulin glargine to standard DM care which was carried out in 12,537 participants with pre-diabetes and newly diagnosed T2DM over a median duration of 6.2 years found neither a beneficial nor detrimental effect of insulin glargine on the development of HF [89]. The study was not designed to specifically study HF, and only 310 of 6264 participants on insulin glargine and 343 of 6273 participants on standard care were hospitalized for HF. The mean HbA1c for the insulin glargine and standard treatment groups for the study were 6.2 and 6.5 %, respectively. Appropriate interventional trials will be necessary to assess the effects of insulin treatment on development and outcomes of HF.

The first clear evidence that anti-hyperglycemic drugs could cause and worsen HF in DM patients was shown with the thiazolidinediones (PPARγ agonists). Pioglitazone and rosiglitazone increase sodium retention by the kidney and cause increased fluid retention which leads to edema and an increase in both the incidence of HF and an increased rate of hospital admissions for HF [90, 91]. These drugs should not be used in patients with T2DM and HF. As noted previously, despite insufficient data about the effect of insulin treatment on outcomes in patients with T2DM and HF, the potential dangers of hypoglycemia are sufficiently serious in this population to limit insulin use.

The effect of DPP-4 inhibitors in patients with T2DM and HF is complicated. The CV outcome trials show that saxagliptin increases hospitalizations for HF in patients with T2DM [92]. The alogliptin trial suggests, but is not definitive, that it too may increase HF hospitalizations [93], while the sitagliptin trial shows no effect on HF morbidity in patients with T2DM [94]. The unresolved question is whether the increase in HF is a class effect and the differences in results due to differences in clinical design of the trials or whether the effect has little to do with DPP-4 inhibition. At the present time, these data suggest that if a DPP-4 inhibitor is to be used in patients with T2DM and HF, sitagliptin would be the one to use.

The LEADER trial with the GLP-1 receptor agonist liraglutide showed no specific effect on HF outcome but did show a decrease in CV mortality in participants with T2DM [95]. Thus, it and perhaps other GLP-1 receptor agonists may be useful in treating the patient with T2DM and HF.

The EMPA-REG CV outcome trial unexpectedly showed that the SGLT-2 inhibitor, empagliflozin, has a unique benefit in decreasing both HF hospitalization and death in participants with T2DM at high risk for CV events [96•]. The mechanism for this effect is unknown but may be related to its effects in causing weight loss, reducing plasma volume, lowering systolic blood pressure, and/or creating a more ketogenic metabolism. A specific outcome trial of empagliflozin in patients with T2DM and HF is needed to verify that the benefits sufficiently outweigh potential side effects before this can be recommended as a preferred treatment. CV safety trials with other SGLT-2 inhibitors such as canagliflozin and dapagliflozin, which will be completed in the next year or two, will show whether the effects on HF outcomes are class effects.

The US FDA has mandated that all new DM drugs must be evaluated for CV safety. This has spawned a large number of completed and ongoing clinical CV trials (Table 3). The trials are primarily designed with the primary endpoint being development of first major CV events defined as CV death, non-fatal myocardial infarction, and non-fatal stroke. HF has not been a major end point, and the studies are not powered for HF outcomes. Nonetheless, data have emerged from some of these trials which have generated significant interest in the indication or contraindication of some of these drugs for the treatment of T2DM patients with HF (Table 3).

**Table 3:** Randomized clinical intervention trials that evaluated the effects of specific anti-glycemic agents on either the development or treatment of heart failure in patients with type 2 diabetes

Intervention trial	Anti-hyperglycemic agent	# of entered patients	Total HF Drug/placebo	HF hospitalization Drug/placebo (# of patients or events/100 pt. yrs.)	HF hospitalization or CV death (# of patients or Events/100 pt. yrs.)	Use in patients with DM and HF
PROactive	Pioglitazone	5238	281/198 *P* = 0.0001	149/108 *P* = 0.007	Death 25/22 *P* = 0.634	Not recommended
RECORD	Rosiglitazone	4447		61/29 *P* = 0.001		Not recommended
SAVOR-TIMI-53	Saxagliptin	16,492		289/228 *P* = 0.007		Not preferred DPP-4 Inhibitor
EXAMINE	Alogliptin	5380		106/89 *P* = 0.15		Not preferred DPP-4 Inhibitor
TECOS	Sitagliptin	14,671		1.07/1.09 *P* = 0.98	2.54/2.50 *P* = 0.74	Preferred DPP-4 Inhibitor
ELIXA	Lixisenatide	6068		1.8/1.9 *P* = 0.75		No detrimental effects
EMPA-REG	Empagliflozin	7020		9.4/14.5 *P* = 0.002	5.7/8.5 *P* < 0.001	Beneficial effects on HF
LEADER	Liraglutide	9340		1.2/1.4 *P* = 0.14		Beneficial in reducing CV deaths but not HF
ORIGIN	Glargine Insulin	12,612		0.85/0.95 *P* = 0.16		Hypoglycemia is a significant risk for HF patients

## Conclusions

Evolving data on HF and DM indicate that DM is present in as many as 50 % of patients presenting with HF. Those patients with DM and HF have a significantly worse outcome than patients with HF and no DM. The person with DM and HF responds less well to medical HF treatments than patients with normal glycemia. The effects of DM on HF is related to the degree of metabolic derangement and are seen even in the pre-diabetic state. Treatment of the patient with DM and HF requires the simultaneous treatment of both the HF and the hyperglycemia. The impact of anti-hyperglycemic agents on the development of HF and its clinical outcomes may open new approaches to treatment. The worldwide incidence of T2DM is increasing in epidemic proportions with the number of patients with DM expected to exceed 640 million by 2040. The majority of patients are developing T2DM in middle age (30 to 60 years) and surviving well into their 70s and 90s. It can be anticipated that the increase in DM and the aging of the population will lead to a marked increase in the number of patients with DM and HF. The increasing number and poor prognosis of DM patients with HF requires the development of new strategies to prevent and to treat this increasing important complication of DM.

## References

[1] Ponikowski P, Voors AA, Anker SD, Bueno H, Cleland JGF, Coats AJS (2016). 2016 ESC guidelines for the diagnosis and treatment of acute and chronic heart failure. Eur J Heart Fail.

[2] Braunwald E (2013). Heart Failure. JCHF.

[3] Braunwald E (2015). The war against heart failure: the Lancet lecture. Lancet.

[4] IDF Diabetes Atlas 7th Edition. International Diabetes Federation. 2015

[5.••] Cavender MA, Steg G, Smith SC, Eagle K, Ohman EM, Goto S (2015). Impact of diabetes mellitus on hospitalization for heart failure, cardiovascular events and death. Outcomes at 4 years from the Reduction of Atherothrombosis for Continued Health (REACH) Registry. Circulation.

[6] Granger CB, McMurray JJ, Yusuf S, Held P, Michelson EL, Olofsson B (2003). Effects of candesartan in patients with chronic heart failure and reduced left-ventricular systolic function intolerant to angiotensin converting enzyme inhibitors: the CHARM-Alternative trial. Lancet.

[7] McMurray JJV, Packer M, Desai AS, Gong J, Lefkowitz MP, Rizkala AR (2014). Angiotensin-Neprilysin inhibition versus Enalapril in heart failure. N Engl J Med.

[8] Kannel WB, Hjortland M, Castelli WP (1974). The role of diabetes in congestive heart failure:the Framingham study. Am J Cardiol.

[9] Nichols GA, Gullion CM, Koro CF, Ephross SA, Brown JB (2005). The incidence of congestive heart failure in type 2 diabetes. Diabetes Care.

[10] Del Cas A, Khan SS, Butler J, Mentz RJ, Bonow RO, Avogaro A (2015). Impact of diabetes on epidemiology, treatment, and outcomes of patients with heart failure. L Am Coll Cardiol HF.

[11] MacDonald MR, Petrie MC, Varyani F, Ostyergren J, Michelson EL, Young JB (2008). Impact of diabetes on outcomes in patients with low and preserved ejection fraction heart failure. Eur Heart J.

[12] Kristensen SL, Preiss D, Jhund PS, Squire I, Cardoso JS, Merkely B (2016). Risk-related to pre-diabetes mellitus and diabetes mellitus in heart failure with reduced ejection fraction. Insights from prospective comparison of ARNI with ACEI to determine global mortality and morbidity in heart failure trial. Circ Heart Fail..

[13] ACC Foundation and AHA task force (Chair Sharon Ann Hunt) (2009). 2009 focused update incorporated into the ACC/AHA 2005 Guidelines for diagnosis and management of heart failure in adults. J Am Coll Cardiol.

[14] New York Heart Association, Inc., Diseases of the Heart and Blood Vessels. Nomenclature and Criteria for Diagnosis, 6th ed. Boston, Little Brown, 1964, p 114

[15] Greenberg BH, Abraham WT, Albert NM, Chiswell K, Clare R, Stough WG (2007). Influence of diabetes on characteristics and outcomes in patients hospitalized with heart failure: A report from the Organized Program to Initiate Lifesaving Treatment in Hospitalized Patients with Heart Failure (OPTIMIZE-HF). Am Heart J.

[16] Wong YW, Thomas L, Sun J-L, McMurray JJV, Krum H, Hernandez AF (2013). Predictors of incident heart failure hospitalizations among patients with impaired glucose tolerance. Circ Heart Fail.

[17] Iribarren C, Karter AJ, Go AS, Ferrara A, Liu JY, Sidney S (2001). Glycemic control and heart failure among adults with diabetes. Circulation.

[18] Lind M, Olsson M, Rosengren A (2012). Svensson A_M, Bounias I, Gudbjornsdottir S: The relationship between glycaemic control and heart failure in 83,021 patients with type 2 diabetes. Diabetologia.

[19.•] Glogner S, Rosengren A, Olsson M, Gudbjornsdottir S, Svensson A-M, Lind M (2014). The association between BMI and hospitalization for heart failure in 83,021 persons with type 2 diabetes: a population-based study from the Swedish National Diabetes Registry. Diabet Med.

[20] Waring ME, Saczynski JS, McManus D, Zacharias M, Lessard D, Gore JM (2011). Weight and mortality following heart failure hospitalization among diabetic patients. Am J Med.

[21] Lang RM, Badano LP, Mor-Avi V, Afilalo J, Armstrong A, Ernande L (2015). Recommendations for cardiac chamber quantification by Echocardiography. J Am Soc Echocardiogr.

[22] Abbate A, Arena R, Abouzaki N, Van Tassell BW, Canada J, Shah K (2015). Heart failure with preserved ejection fraction: refocusing on diastole. Int J Cardiol.

[23] Paulus WJ, Tschope C, Sanderson JE, Rusconi C, Flachskampf FA, Rademakers FE (2007). How to diagnose diastolic heart failure: a consensus statement on the diagnosis of heart failure with normal left ventricular ejection fraction by heart failure and echocardiography associations of the European society of cardiology. Eur Heart j.

[24] Velagaleti RS, Gona P, Pencina MJ, Aragam J, Wang TJ, Levy D (2014). Left ventricular hypertrophy patterns and incidence of heart failure with preserved versus reduced ejection fractions. Am J Cardiol.

[25] Borbely A, Papp Z, Edes I, Paulus WJ (2009). Molecular determinants of heart failure with normal left ventricular ejection fractions. Pharmacological Reports.

[26] Kemp CD, Conte JV (2012). The pathophysiology of heart failure. Cardiovascular Pathology.

[27] Mentz RJ, O’Connor CM (2016). Pathophysiology and clinical evaluation of acute heart failure. Nat Rev Cardiol.

[28] Haydock PM, Cowie MR (2010). Heart failure: classification and pathophysiology. Medicine.

[29] Volpe M, Carnovali M, Mastromarino V (2016). The natriuretic peptides system in the pathophysiology of heart failure: from molecular basis to treatment. Clinical Science.

[30] Von Lueder TG, Krum H (2015). New medical therapies for heart failure. Nat Rev Cardiol.

[31] The Task Force on diabetes, pre-diabetes and cardiovascular diseases of the European Society of Cardiology. ESC Guidelines on diabetes. pre-diabetes and cardiovascular diseases developed in collaboration with the EASD. Eur Heart J. 2013;34:3035–87. 10.1093/eurheartj/eht108 23996285

[32] 2013 ACCF/AHA Guideline for the management of heart failure. J Am Coll Cardiol. 2013;62(16):e147–e239. 10.1016/j.jacc.2013.05.019 23747642

[33] Sharma K, Kass DA (2014). Unmet needs in cardiovascular science and medicine. Circ Res.

[34] Hoenig MR, Bianchi C, Rosenzweig A, Sellke EW (2008). The cardiac microvasculature in hypertension, cardiac hypertrophy and diastolic heart failure. Curr Vasc Pharmacol.

[35] Kawata T, Daimon M, Miyazaki S, Ichikawa R, Maruyama M, Chiang S-J (2015). Coronary microvascular function is independently associated with left ventricular filling pressure in patients with type 2 diabetes mellitus. Cardiovasc Diabetol.

[36] Owan TE, Hodge DO, Herges RM, Jacobsen SJ, Roger VL, Redfield MM (2006). Trends in prevalence and outcome of heart failure with preserved ejection fraction. N Engl J Med.

[37] Aquilar D, Deswal A, Ramasubbu K, Mann DL, Bozkurt B (2010). Comparison of patients with heart failure and preserved ejection fraction among those with versus without diabetes mellitus. Am J Cardiol.

[38] Cubbon RM, Adams B, Rajwani A, Mercer BN, Patel PA, Gheradi G (2013). Diabetes mellitus is associated with adverse prognosis in chronic heart failure of ischaemic and non-ischaemic aetiology. Diab Vasc Dis Res.

[39.••] Bando YK, Murohara T (2014). Diabetes-related heart failure-Does diabetic cardiomyopathy exist?. Circulation Journal.

[40] Amaral N, O’okonko DO (2015). Metabolic abnormalities of the heart in type 2 diabetes. Diabetes & Vascular Disease Research.

[41] Skali H, Shah A, Gupta DK, Cheng S, Claggett B, Liu J (2015). Cardiac structure and function across the glycemic spectrum in elderly men and women free of prevalent heart disease. The Atherosclerosis Risk in Community Study. Circ Heart Fail.

[42] Bugger H, Able ED (2014). Molecular mechanisms of diabetic cardiomyopathy. Diabetologia.

[43] Miki T, Yuda S, Kouzu H, Miura T (2013). Diabetic cardiomyopathy: pathophysiology and clinical features. Heart Fail Rev.

[44] Wang ZV, Li DL, Hill JA (2014). Heart failure and loss of metabolic control. J Cardiovasc Pharmacol.

[45] Bertoni AG, Tsai A, Kasper EK, Brancati FL (2003). Diabetes and idiopathic cardiomyopathy. Diabetes Care.

[46] The Diabetes Control and Complications Trial Research Group (1993). The effect of intensive treatment of diabetes on the development and progression of long-term complications in insulin-dependent diabetes mellitus. N Engl J Med.

[47] Mosterd A, Hoes AW (2007). Clinical epidemiology of heart failure. Heart.

[48] Ahmed A, Aronow WS, Fleg JL (2006). Higher New York Heart Association classes and increased mortality and hospitalizations in heart failure patients with preserved left ventricular function. Am Heart J.

[49] Gotsman I, Shauer A, Lotan C, Keren A (2014). Impaired fasting glucose: a predictor of reduced survival in patients with heart failure. Eur J Heart Fail.

[50] Flores-Le Roux J, Comin J, Pedro-Botet J, Benaiges D, Puig-de Dou J, Chillaron JJ (2011). Seven-year mortality in heart failure patients with undiagnosed diabetes: an observational study. Cardiovascular Diabetology.

[51.•] Helfand BKI, Maselli NJ, Lessard DM, Yarzebski J, Gore JM, McManus DD (2015). Elevated serum glucose levels and survival after acute heart failure: A population-based perspective. Diabetes & Vascular Disease Research.

[52] Mesotten d, Van den Berghe G (2012). Glycemic targets and approaches to management of the patient with critical illness. Curr Diab Rep.

[53] Pocock SJ, Ariti CA, McMurray JJV, Maggioni A, Keber L, Squire IB (2013). Predicting survival in heart failure: a risk score based on 39,372 patients from 30 studies. Eur Heart J.

[54] Levy WC, Mozaffarian D, Linker DT, Sutradhar SC, Anker SD, Cropp AB (2006). The Seattle Heart Failure Model. Prediction of survival in heart failure. Circulation.

[55] Garg R, Yusuf S (1995). Overview of randomized trials of angiotensin converting enzyme inhibitors on mortality and morbidity in patients with heart failure. Collaborative Group on ACE inhibitor trials. JAMA.

[56] CONSENSUS trial study group (1987). Effects of enalapril on mortality in severe congestive heart failure. Results of the Cooperative North Scandinavian Enalapril Survival Study (CONSENSUS). N Engl J Med.

[57] SOLVD investigators (1991). Effect of enalapril on survival in patients with reduced left ventricular ejection fractions and congestive heart failure. N Engl J Med.

[58] SOLVD investigators (1992). Effect of enalapril on mortality and the development of heart failure in asymptomatic patients with reduced left ventricular ejection fractions. N Engl J Med.

[59] Konstam MA, Neaton JD, Dickstein K, Drexler H, Komaida M, Martinez FA (2009). HEAAL investigators: Effects of high dose versus low dose losartan on clinical outcomes in patients with heart failure (HEAAL study): a randomized, double-blind trial. Lancet.

[60] Cohn JN, Tognoni G, Valsartan Heart Failure Trial Investigators (2001). A randomized trial of the angiotensin receptor blocker valsartan in chronic heart failure. N Engl J Med.

[61] Maggioni AP, Anand I, Gottlieb SO, Latini R, Tognoni G, Cohn JN (2002). Effects of valsartan on morbidity and mortality in patients with heart failure not receiving angiotensin-converting enzyme inhibitors. J Am Coll Cardiol.

[62] McMurray JJV, Ostergren J, Swedberg K, Granger CB, Held P, Michelson EL (2003). Effects of candesartan in patients with chronic heart failure and reduced left-ventricular systolic function taking angiotensin converting enzyme inhibitors: the CHARM-Added trial. Lancet.

[63] McMurray JJV, Krum H, Abraham WT, Dickstein K, Kober AV, Desai AS (2016). Aliskiren, enalapril, or aliskiren and enalapril in heart failure. N Engl J Med.

[64] The cardiac insufficiency bisoprolol study II (CIBIS-II): a randomized trial. Lancet 1999, 353:9-13. 10023943

[65] Effect of metoprolol CR/XL in chronic heart failure: Metoprolol CR/XL randomized intervention trial in congestive heart failure (MERIT-HF). Lancet 1999, 353:2001-2007. 10376614

[66] Packer M, Coats AJ, Fowler MB, Katus HA, Krum H, Mohacsi P (2001). Effects of carvedilol on survival in severe chronic heart failure. N Engl J Med.

[67] Packer M, Fowler MB, Roecker EB, Coats AJ, Katus HA, Krum H (2002). Effect of carvedilol on the morbidity of patients with severe chronic heart failure: results of the carvedilol prospective randomizrd cumulative survival (COPERNICUS) study. Circulation.

[68] Pitt B, Zannad F, Remme WJ, Cody R, Castaigne A, Perez A (1999). The effect of spironolactone on morbidity and mortality in patients with severe heart failure. Randomized Aldactone Evaluation Study Investigators. N Engl J Med.

[69] Zannad F, McMurray JJ, Krum H, van Veldhuisen DJ, Swedberg K, Shi H (2011). Eplerenone in patients with systolic heart failure andmild symptoms. (EMPHASIS-HF Study). N Engl J Med.

[70] Zois NE, Bartels ED, Hunter I, Kousholt BS, Olsen LH, Goetze JP (2014). Natriuretic peptides in cardiometabolic regulation and disease. Nat Rev Cardiol.

[71] Singh JSS, Lang CC (2015). Angiotensin receptor-neprilysin inhibitors: clinical potential in heart failure and beyond. Vascular Health and Risk Management.

[72] Swedberg K, Komaida M, Bohm M, Borer JS, Ford I, Dubost-Brama A (2010). Ivabradine and outcomes in chronic heart failure (SHIFT): a randomized placebo controlled trial. Lancet.

[73] Bohm M, Robertsom M, Ford I, Borer JS, Komajda M, Kindermann I (2015). Influence of cardiovascular and noncardiovascular co-morbidities on outcomes and treatment effect of heart rate reduction with Ivabradine in stable heart failure (fom the SHIFT trial). Am J Cardiol.

[74] The Digitalis Investigation Group (1997). The effect of digoxin on mortality and morbidity in patients with heart failure. N Engl J Med.

[75] Ahmed A, Rich MW, Fleg JL, Zile MR, Young JB, Kitzman DW (2006). Effects of digoxin on morbidity and mortality in diastolic heart failure. Circulation.

[76] Yusuf S, Pfeffer MA, Sedberg K, Granger CB, Held P, McMurray JJV (2003). Effects of candesartan in patients with chronic heart failure and preserved left-ventricular ejection fraction: the CHARM Preserved Trial. Lancet.

[77] Massie BM, Carson PE, McMurray JJ, Komajda M, McKelvie R, Zile MR (2008). Irbesartan in patients with heart failure and preserved ejection fraction. N Engl J Med.

[78] Solomon SD, Zile M, Pieske B, Voors A, Shah A, Kraigher-Krainer E (2012). The angiotensin receptor neprilysin inhibitor LCZ696 in heart failure with preserved ejection fraction: a phase 2 double-blind randomized controlled trial. Lancet.

[79] Turnbull FM, Abraira C, Anderson RJ, Byington RP, Chalmers JP, Duckworth WC (2009). Intensive glucose control and macrovascular outcomes in type 2 diabetes. Diabetologia.

[80] Sud M, Wang X, Austin PC, Lipscombe LL, Newton GE, Tu JV (2015). Presentation blood glucose and death, hospitalization and future diabetes risk in patients with acute heart failure syndromes. Eur Heart J.

[81] Maggioni AP, Greene SJ, Fonarow GC, Bohm M, Zannad F, Solomon SD (2013). Effect of aliskiren on post-discharge outcomes among diabetic and non-diabetic patients hospitalized for heart failure: insights from the ASTRONAUT trial. Eur Heart J.

[82] Sarma S, Mentz RJ, Kwasny MJ, Fought AJ, Huffman M, Subacius H (2013). Association between diabetes mellitus and post-discharge outcomes in patients hospitalized with heart failure: findings from the EVEREST trial. Eur j Heart Fail.

[83] Aguilar D, Bozkurt B, Ramasubbu K, Deswal A (2009). Relationship of hemoglobin A1C and mortality in heart failure. J Am Coll Cardiol.

[84] Tomova GS, Nimbal V, Horwich TB (2012). Relation between hemoglobin A1C and outcomes in heart failure patients with and without diabetes mellitus. Am J Cardiol.

[85] Eshaghian S, Horwich TB, Fonarow GC (2006). An unexpected inverse relationship between HbA1c levels and mortality in patients with diabetes and advanced systolic heart failure. Am Heart J.

[86] Aguilar D, Chan W, Bozkurt B, Ramasubbu K, Deswal A (2011). Metformin use and mortality in ambulatory patients with diabetes and heart failure. Circ Heart Fail.

[87] Tahrani AA, Varughese GI, Scarpello JH, Hanna FW (2007). Metformin, heart failure, and lactic acidosis: is metfromin absolutely contraindicated?. BMJ.

[88] Smooke S, Horwich TB, Fonarow GC (2005). Insulin-treated diabetes is associated with a marked increase in mortality in patients with advanced heart failure. Am Heart J.

[89] Gerstein HC, Bosch J, Dagenais GR, Diaz R, Jung H, Maggioni AP (2012). Basal insulin and cardiovascular and other outcomes in dysglycemia. N Engl J Med.

[90] Dormandy JA, Charbonnel B, Eckland DJA, Erdmann E, Massi-Benedetti M, Moules IK (2005). Secondary prevention of macrovascular events in patients with type 2 diabetes in the PROactive (PROspective pioglitazone Clinical Trial in macrovascular Events): a randomized controlled tria. Lancet.

[91] Home PD (2009). Pocock Sj, Beck-Nielsen H, Curtis PS, Gomis R, Hanefeld M, Jones MP, Komajda M: Rosiglitazone evaluated for cardiovascular outcomes in oral agent combination therapy for type 2 diabetes (RECORD): a multicenter, randomized, open-label trial. Lancet.

[92] Scirca BM, Bhatt DL, Braunwald E, Steg PG, Davidson J, Hirshberg B, Ohman P, Frederich R, Wiviott SD, Hoffman EB, Cavender MA, Udell JA, Desai NR, Mozensen O, McGuire DK, Pay KK, Leiter LA, Raz I: Saxaliptin and cardiovascular outcomes in patients with type 2 diabetes mellitus. New Engl J Med. 2013;369:1317–26.

[93] White WB, Cannon CP, Heller SR, Nissen SE, Bergenstal RM, Bakris GL (2013). Alogliptin after acute coronary syndrome in patients with type 2 diabetes. N Engl J Med.

[94] Green JB, Bethel A, Armstrong PW, Buse JB, Engel SS, Garg J (2015). Effect of Sitagliptin on cardiovascular outcomes in type 2 diabetes. N Engl J Med.

[95] Marso SP, Daniels GH, Brown-Frandsen K, Kristensen P, Mann JFE, Nauck MA (2016). Liraglutide and cardiovascular outcomes in type 2 diabetes. N Engl J Med.

[96.•] Zinman B, Wanner C, Lachin JM, Fitchett D, Bluhmki E, Hantel S (2015). Empagliflozin, cardiovascular outcomes and mortality in type 2 diabetes. N Engl J Med.

